# Potential of smartwatch touchscreen glass for electron paramagnetic resonance dosimetry in radiological emergencies

**DOI:** 10.3389/fpubh.2025.1739627

**Published:** 2026-01-20

**Authors:** Jae Seok Kim, Byeong Ryong Park, Byeong Min Lee, Chanwoo Park, MinSeok Park, Seokwon Yoon, Yejin Kim, Kihoon Kim, Minsu Cho, Kyu Seok Cho, HyoJin Kim

**Affiliations:** 1 National Radiation Emergency Medical Center, Korea Institute of Radiological and Medical Sciences, Seoul, Republic of Korea; 2 Dongnam Institute of Radiological & Medical Sciences, Busan, Republic of Korea

**Keywords:** electron paramagnetic resonance, radiological emergencies, radiological characteristics, retrospective dosimetry, smartwatch touchscreen glass

## Abstract

**Introduction:**

In radiological emergencies, retrospective dosimetry is essential for estimating absorbed radiation doses when conventional dosimetric data are unavailable. Various human-derived tissues and surrounding materials have been used for dose assessment using electron paramagnetic resonance (EPR) techniques. However, because the retrospective dosimetric materials have inherent limitations, dose assessment should reasonably be determined through the combined application of various retrospective dosimetry methods. With the increasing use of smartwatches for health monitoring and fashion, touchscreen glass on smartwatches has a potential as a material for retrospective dosimetry.

**Methods:**

The radiological characteristics of smartwatch touchscreen glass (STG) were evaluated for its potential role in public health emergency preparedness during radiological incidents. STGs samples extracted from several smartwatch models were evaluated for their radiological characteristics, including background signal, mechanically induced signal, light-induced signals, radiation-induced signal (RIS), time stability of RIS, dose–response relationship, ultraviolet (UV) effects on RIS, thermal and pretreatment effects on RIS, and the minimum detectable dose.

**Results:**

Among the tested samples, STG-4, derived from the Mi Band series produced by Xiaomi, demonstrated the most suitable performance in various radiological characteristics. Based on the radiological characteristics, a preliminary STG-EPR dosimetry protocol was established, and a blind test was performed using E_n_ score analysis under laboratory conditions. The E_n_ scores, calculated from the evaluated and reference doses, were within ±1, satisfying the acceptance criterion specified by ISO 13528.

**Discussion:**

STG-4 was confirmed as a potential material for application in EPR dosimetry through the blind test, demonstrating the feasibility of achieving rapid and reliable dose assessment using STG. Because the UV effects on RIS and the material composition of the STGs vary by manufacturer and smartwatch version, further research is recommended to optimize their use in radiation emergency response and public health protection.

## 1 Introduction

Radiation technologies are widely applied in various fields, including medicine, industry, agriculture, research, and environmental management. However, the increasing application of radiation technologies raises the potential for radiological accidents resulting from equipment malfunctions, human error, or procedural lapses. In such incidents, retrospective dosimetry is essential for estimating the absorbed dose when conventional dosimetric data are unavailable or insufficient (1). Among various retrospective dosimetry techniques, electron paramagnetic resonance (EPR) dosimetry has attracted considerable attention due to its ability to detect radiation-induced signals in biological and physical samples, such as tooth enamel, fingernails, bones, and personal belongings. Tooth enamel is considered a representative material for EPR dosimetry because of its high dose–response and signal stability (2–5). However, its collection is invasive and impractical in emergency situations (6–8). Fingernails are useful for assessing doses of hand-localized exposures, which are common in radiological accidents, but their radiation-induced signals are sensitive to environmental factors such as humidity and temperature (9–16). To overcome the limitation of biological samples, various components of personal belongings were evaluated for application in EPR dosimetry (17–21). Among these components, smartphone touchscreen glass is one of the representative materials used for retrospective dosimetry. Wu et al. (18) demonstrated the feasibility of a watch glass for EPR dosimetry in radiological emergencies, while Fattibene et al. (19) conducted an intercomparison study using smartphone touchscreen glass by applying the previously established radiological characteristics of common glass materials. Juniewicz et al. (20) analyzed the effects of sunlight and UV exposure on the EPR signal and, more recently, Marciniak et al. (22) investigated and classified the radiological characteristics of smart device glasses for retrospective dosimetry. The use of smartwatches among various personal belongings has been steadily increasing for health monitoring and fashion purposes among various personal belongings. Moreover, most smartwatches are relatively inexpensive compared to smartphones and are typically worn on a fixed part of the body, making them suitable candidates for dose assessment. In this study, the applicability of dose assessment was evaluated using the radiological characteristics of smartwatch touch glass (STG) for radiological emergencies. The radiological characteristics of STG examined in this study include the background signal (BKS), radiation-induced signal (RIS), mechanically induced signal (MIS), light-induced signal (LIS), time stability of RIS, dose response, ultraviolet (UV) effect on RIS, thermal and pretreatment effects on RIS, and minimum detectable dose (MDD) (3). Based on these characteristics, a preliminary protocol for STG-based EPR dosimetry was developed, and its potential was verified through a blind test conducted under laboratory conditions (23).

## 2 Materials and methods

EPR measurements were performed using a Bruker E500 EPR spectrometer (Bruker, Germany) equipped with a Bruker SHQE 4122 cavity resonator operating in the X-band (8–12 GHz). All measurements were performed under ambient conditions with a relative humidity of 35 ± 2% and a room temperature of 23 ± 1 °C. The central magnetic field was set at 351 mT. The parameters for EPR measurement were as follows: sweep width (20 mT), receiver gain (30 dB), modulation amplitude (0.4 mT), microwave frequency (9.85 GHz), number of scans (10), sweep time (30.72 s), and microwave power (1.002 mW). For the analysis, quartz thin-walled EPR tubes (4 mm in diameter) were utilized to accommodate the STG samples, prepared in either powdered or bulk form. The powdered samples had particle sizes ranging from 100–1,000 μm and a weight of 100 mg, while the bulk (anisotropic) STG samples had a particle sizes of 3,000–5,000 μm and a similar weight of 100 mg. To ensure the reliability, each sample was measured at least three times, with the tube shaken between measurements to account for anisotropy-induced variations in the results. The intensity of the EPR signal was determined from the peak-to-peak amplitude of the spectrum.

### 2.1 STG

To evaluate the radiological characteristics of STG, four smartwatch models were selected. According to market data from the first quarter of 2025, Apple held the largest share of the global smartwatch market (36%), followed by Samsung (10%), Huawei (7%), and Xiaomi (5%) (24). One representative model was selected from each manufacturer, and multiple identical models were used to evaluate the radiological characteristics. The analysis results of the material composition and physical specification of STG are shown in Table 1. The material composition was analyzed using inductively coupled plasma optical emission spectrometry and wavelength-dispersive X-ray fluorescence spectrometry at the Korea Polymer Testing & Research Institute. The radiological characteristics of STGs were not evaluated according to the molecular structure or material composition.

**Table 1:** Information of STGs according to manufacturer.

Manufacturer	Samsung	Apple	Huawei	Xiaomi
Appearance	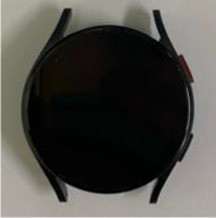	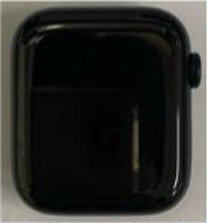	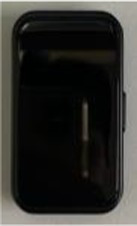	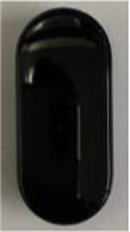
Sample label	STG-1	STG-2	STG-3	STG-4
Type of STG	Sapphire crystal	Ion-X strengthened glass	Polycarbonate	2.5 D tempered glass
Material composition	Si(26.90%) Al(10.40%) Na(0.008%) K(0.004%) Zr(0.003%)	Si(27.70%) Al(11.00%) Na(2.38%) Li(1.40%) Zr(0.77%)	Si(24.40%) Al(6.70%) Na(4.94%) K(3.58%) Zr(0.5%)	Si(24.83%) Al(6.74%) Na(5.21%) K(3.68%) Zr(0.39%)

### 2.2 Classification of EPR spectra in STG

To apply STG in dose assessment, EPR spectrum should first be analyzed. According to previous studies (25–27), the EPR spectra of glass are influenced by various external factors. The BKS represents the intrinsic EPR spectrum obtained in the absence of external pretreatment or irradiation. The MIS corresponds to the EPR spectrum mechanically induced in STG, resulting from the mechanical stress of the samples. Additionally, LIS are generated by exposure to UV light, and RIS refer to signals induced by ionizing radiation. The BKS, LIS, and RIS were measured using powdered samples, while the MIS was measured using both bulk and powdered samples. The BKS was obtained by separating the STGs from smartwatches and performing annealing at 200 °C for 1 h using a dry oven (JEIO TECH, Republic of Korea) (26). Annealing was conducted to determine the BKS by removing potential non-specific signals induced during the manufacturing and transportation of the smartwatches. To evaluate the MIS, each STG sample was first measured in bulk form and then crushed into powder for repeated measurements. For accurate comparison, mass correction was applied to the MIS to minimize distortion caused by sample loss. The LIS and RIS were analyzed based on EPR spectral changes following UV light and gamma-ray stimulation. External stimulation of UV light and radiation was performed using a UV curing system (Skycares, Republic of Korea) and a BioBeam 8,000 irradiator (STS Steuerungstechnik & Strahlenschutz GmbH, Germany). The wavelength and fluence of the UV light were 365 nm and 2,905 kJ/m^2^, respectively. Gamma irradiation was conducted using the BioBeam 8,000 irradiator at a dose of 20 Gy from a ^137^Cs radioisotope source.

### 2.3 Time stability of RIS

In retrospective dosimetry, it is often challenging to immediately assess the absorbed dose of exposed personal belongings after radiological incidents. Therefore, evaluating the time stability of the RIS is essential to ensure accurate dose assessment. The STG samples were irradiated with a radiation dose of 20 Gy, and their EPR spectra were monitored over 4 months. The samples were stored under controlled conditions at 23 ± 1 °C and 35 ± 2% relatively humidity, while being continuously exposed to visible light. The influence of visible light on RIS was assumed to be insignificant in this study.

### 2.4 Dose response

Because EPR dosimetry relies on regression analysis derived from dose–response curves, it is necessary to evaluate the response of STG samples to different radiation doses. The EPR measurements for dose response were therefore conducted using STG samples irradiated with radiation doses of 0, 5, 10, 15, and 20 Gy.

### 2.5 Light effect on RIS

To evaluate the variation in RIS caused by UV light exposure, 20 Gy-irradiated STG samples were assessed according to the amount of UV light exposure. The UV light consisted of approximately 95% UVA and 5% UVB, as UVC is completely absorbed by the stratospheric ozone layer in real situation (28). Using a UV light meter (LT Lutron, Republic of Korea) to measure natural sunlight, the intensity of UVB irradiation was found to be approximately one-hundredth that of UVA. Therefore, UVA was used as the representative component of actual UV light to evaluate the light effect on RIS. To assess the effect of UV light exposure time, it was assumed that the samples were exposed to an irradiance of 2.5 mW/cm^2^, a daily sunlight exposure of 12 h, and the variation in RIS was compared with the time stability of RIS.

### 2.6 Thermal effect on RIS

Smartwatches can be exposed to a variety of thermal conditions. Engin et al. (29) investigated the thermal effect on RIS under different temperature conditions showing that the RIS in window glass exhibited signal fading depending on the annealing and storage temperatures. STG samples were evaluated at various temperatures. To evaluate the thermal stability of RIS, the samples were subjected to thermal exposure in a dry oven at 50 °C for 1, 8, and 24 h. Additionally, to assess the effect of relatively high-temperature exposure, samples were exposed at 100 °C and 200 °C for 1 h.

### 2.7 Pretreatment effect on RIS

Pretreatment is conducted to improve the accuracy of dose assessment and the efficiency of EPR measurement by reducing distortion of the EPR spectrum. In STG-EPR dosimetry, pretreatment involves chemical processing using a solution to remove surface impurities and adhesives, as well as preparing the sample in powdered form to improve measurement efficiency. Bulk samples were used to minimize sample loss during chemical treatment. Additionally, the samples were shaken between measurements, and repeated measurements were performed to reduce measurement variability caused by sample anisotropy. Chemical processing consisted of treating bulk STG fragments for 5 min with three cleaning solutions: distilled water, 99% ethanol (DUKSAN, Republic of Korea), and a commercial sticker remover (NABAKEM, Republic of Korea).

### 2.8 Minimum detectable dose

The minimum detectable dose (MDD) was determined according to the guidelines outlined in ISO 13304-2 (3). Establishing distinct criteria to differentiate between the BKS and RIS was crucial for accurately determining the MDD. An EPR signal was considered RIS when its intensity exceeded three times the standard deviation of the BKS, ensuring statistical significance (30).

### 2.9 Blind test of the preliminary STG-EPR dosimetry protocol

A blind test was conducted under laboratory conditions to assess the potential of the preliminary STG-EPR dosimetry protocol designed based on the radiological characteristics of STG. Two smartwatches were irradiated with different radiation doses. To validate the protocol under a realistic post-accident scenario, the blind test was designed based on the assumption that the dose assessment would be conducted 5 d after the exposure and that the device was worn and used normally during this latency period. To simulate the environmental factors encountered during this period, the smartwatches were subjected to thermal exposure in a dry oven at 50 °C for 4 h and exposed to UV light using a UV curing system for 10 s after irradiation. Subsequently, the irradiated samples were stored under ambient conditions (relative humidity: 35 ± 2%, temperature of 23 ± 1 °C) for 5 d. STG samples were exposed to UV light at an intensity of 3,228 mW/cm^2^, corresponding to a cumulative exposure of 968 kJ/m^2^. This exposure energy is equivalent to sunlight UV exposure at 2.5 mW/cm^2^ over a continuous duration of 0.98 d. After 5 days, the STGs were extracted from both devices and processed according to the established protocol. The evaluated doses were compared with the reference doses as follows:


(1) $$E_{n}=\frac{x-X}{\sqrt{{U_{x}}^{2}+{U_{X}}^{2}}}$$

where *x* represents the evaluated dose in the STG, *X* represents the reference dose, *U_x_* is the expanded uncertainty of the evaluated dose in the STG, and *U_X_* represents the expanded uncertainty of the reference dose. The *E_n_* scores were calculated using Equation 1 based on ISO 13528(2022) (23).

## 3 Results and discussion

### 3.1 EPR spectra in STG

**Figure 1:**
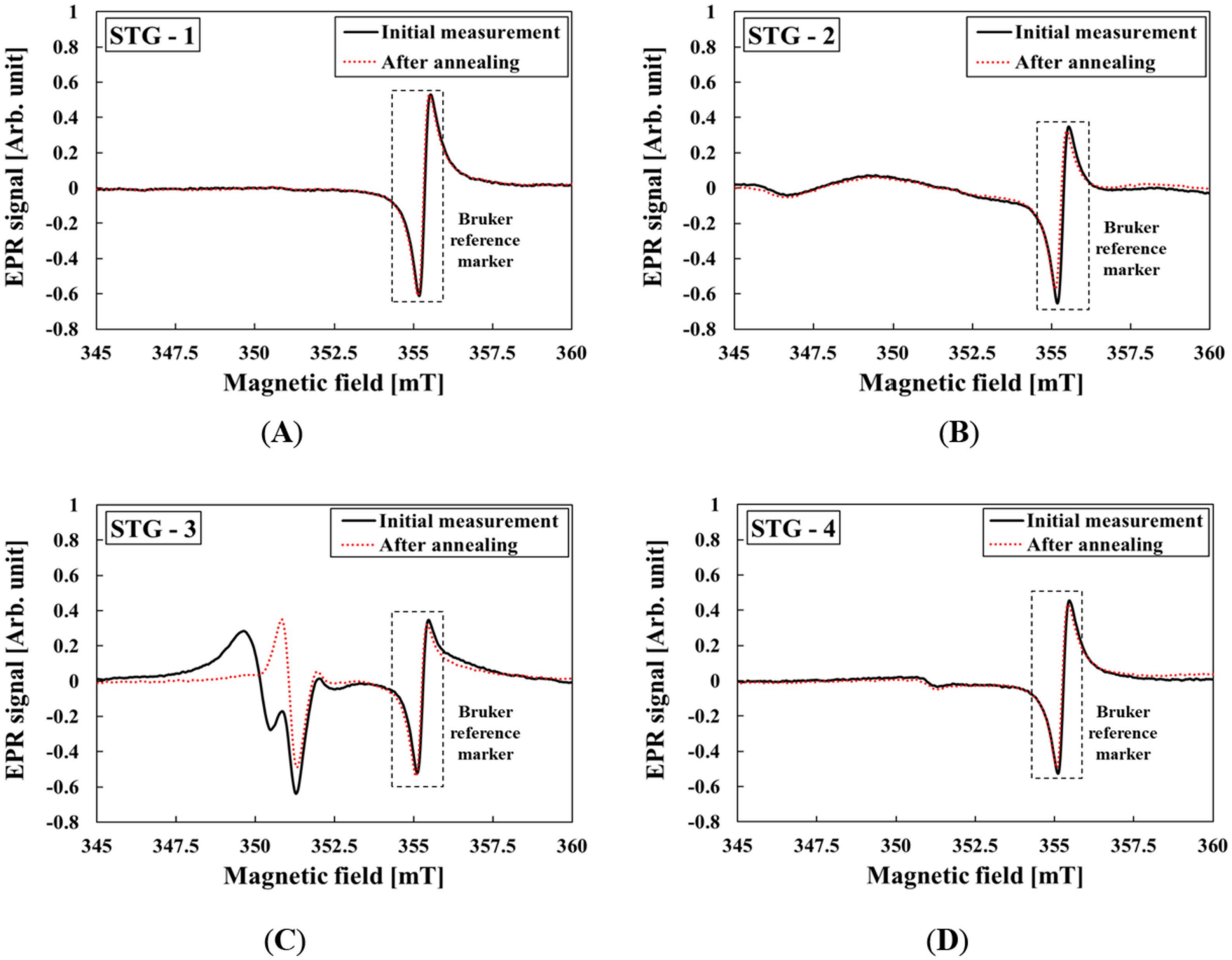
EPR spectra of BKS from STG samples before and after annealing: **(A)** STG-1, **(B)** STG-2, **(C)** STG-3, and **(D)** STG-4.

The EPR spectra of the different types of STGs were obtained under the effects of annealing, mechanical stress, UV light exposure, and irradiation. The BKS was evaluated from powdered samples after annealing, and the results of the BKS in the STGs are shown in Figure 1. The EPR spectra obtained after annealing were determined as the BKS. STG-1, STG-2, and STG-4 exhibited identical EPR spectra before and after annealing. However, STG-3 showed spectral changes due to annealing. Consequently, the intrinsic signals remaining after thermal stabilization were used as the baseline for dose assessment. The subsequent evaluation of the radiological characteristics of the STGs was conducted using the annealed STG samples.

**Figure 2:**
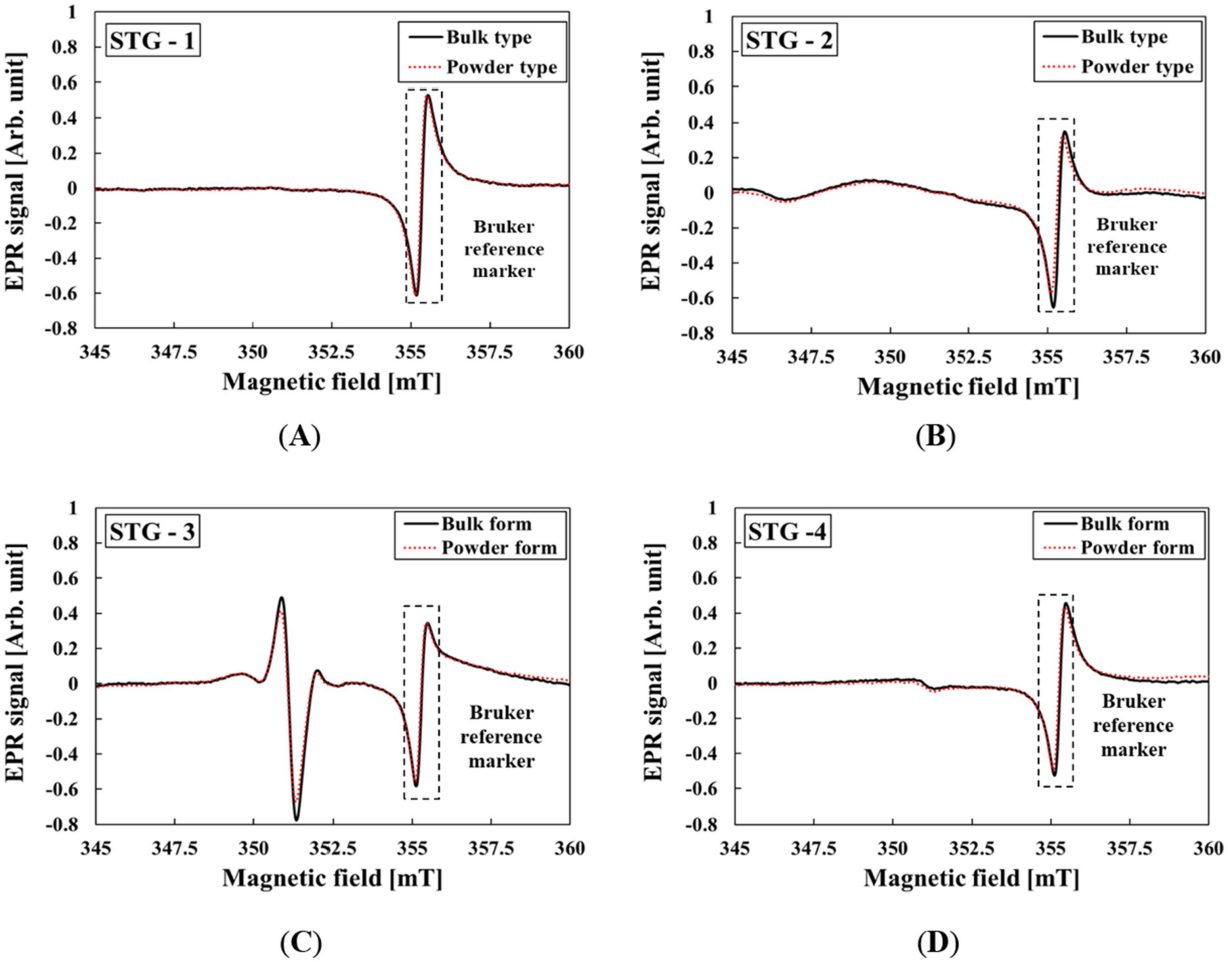
EPR spectra of MIS from STG samples before and after mechanical stress: **(A)** STG-1, **(B)** STG-2, **(C)** STG-3, and **(D)** STG-4.

Figure 2 shows the results of STG samples crushed from bulk into powder form. MIS was not observed in the EPR spectra of the STGs. The slight EPR spectral differences observed in Figure 2 are attributed variations in the cavity Q-factor resulting from the difference in packing factor between the bulk and powdered samples (31). Additionally, no specific signals induced by mechanical stress were detected in the STGs.

**Figure 3:**
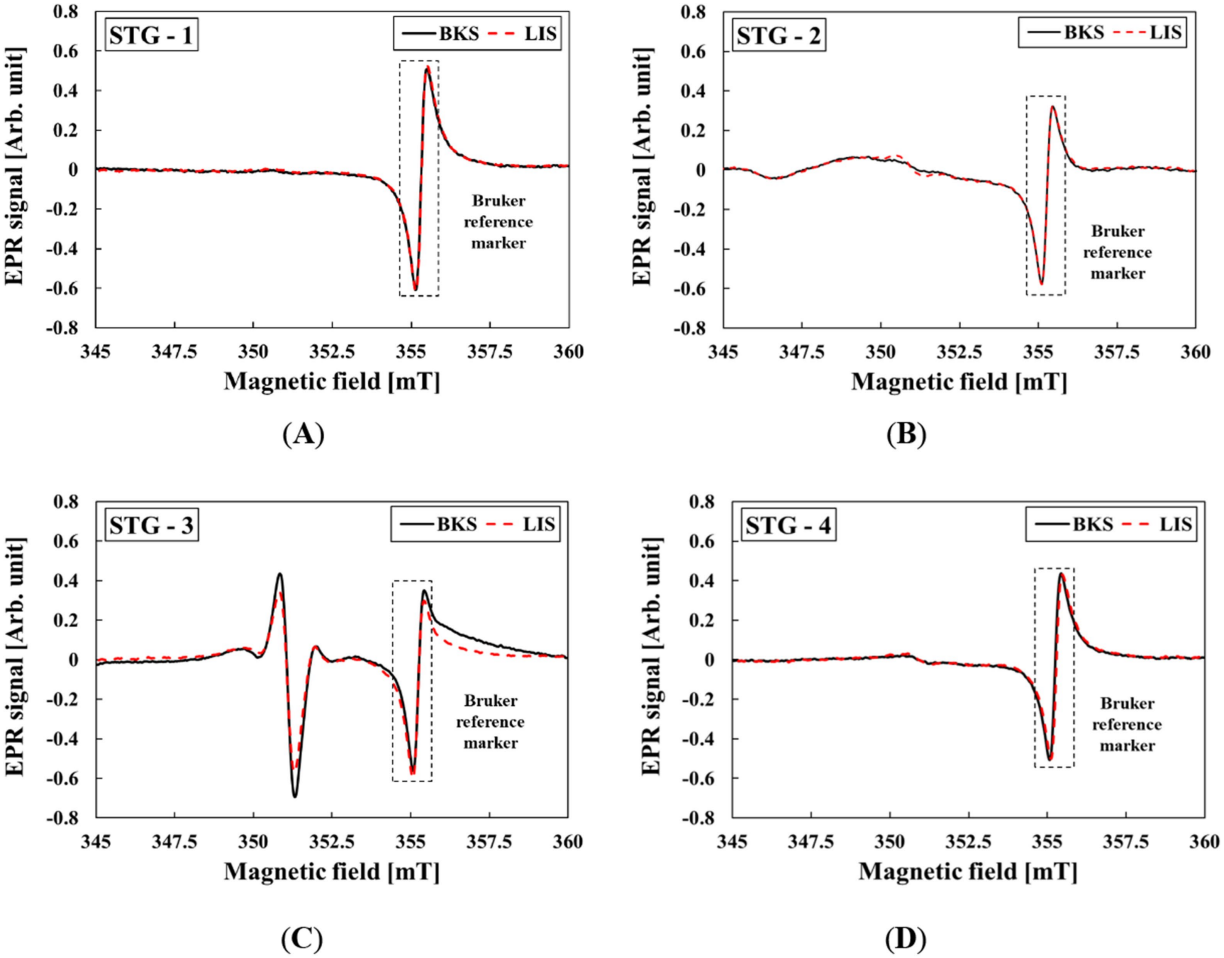
EPR spectra of LIS from STG samples before and after UV light exposure: **(A)** STG-1, **(B)** STG-2, **(C)** STG-3, and **(D)** STG-4.

The changes in EPR spectra were further evaluated following UV light exposure. Figure 3 shows the result of LIS in STGs. In STG-2, slight variations in the EPR spectrum were observed; however, after a few minutes, repeated measurements were consistent with the EPR spectrum of BKS. Consequently, LIS was not detected in STG-1, STG-3, or STG-4.

**Figure 4:**
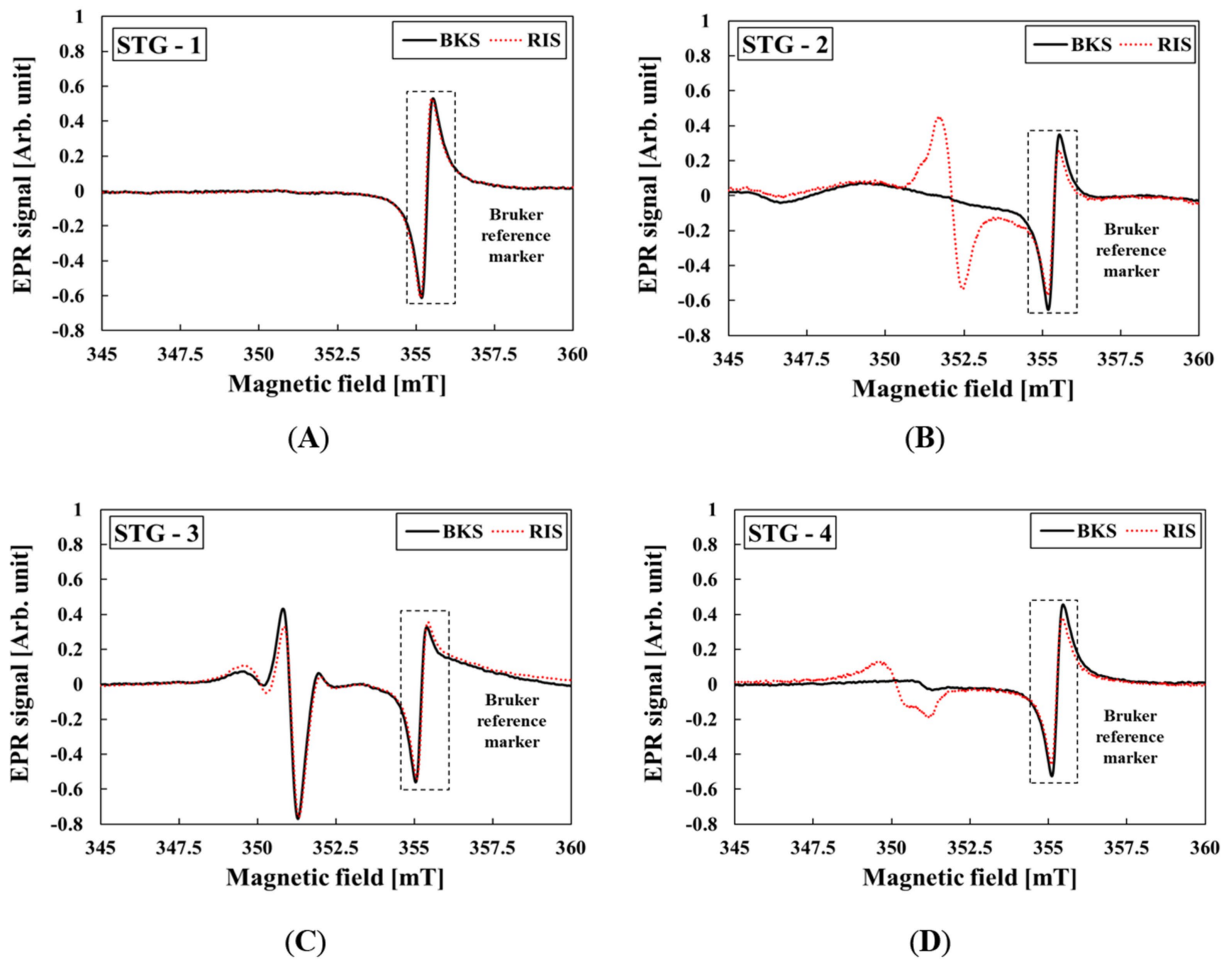
EPR spectra of STG samples according to four smartwatch models before and after irradiation with 20 Gy: **(A)** STG-1, **(B)** STG-2, **(C)** STG-3, and **(D)** STG-4.

A clear distinction between BKS and RIS is essential for the practical application of STG in dose assessment. Figure 4 shows the EPR spectra obtained for the four types of STGs following irradiation with 20 Gy.

The g-factor of the STG samples were evaluated to be in the range of 1.998–2.008. The observed g-factors are characteristics of intrinsic radiation-induced defect centers in silicate glasses (32). The proportion of RIS in each STG sample was evaluated relative to the overall spectrum, including both BKS and RIS for the following smartwatch models: STG-1 (0.31%), STG-2 (86.02%), STG-3 (14.11%), and STG-4 (79.89%). STG-2, STG-3, and STG-4 exhibited distinct RIS, whereas STG-1 showed no detectable EPR spectra, indicating limited applicability for retrospective dosimetry. Therefore, STG-1 was excluded from subsequent analyses.

### 3.2 Time stability of RIS

**Figure 5:**
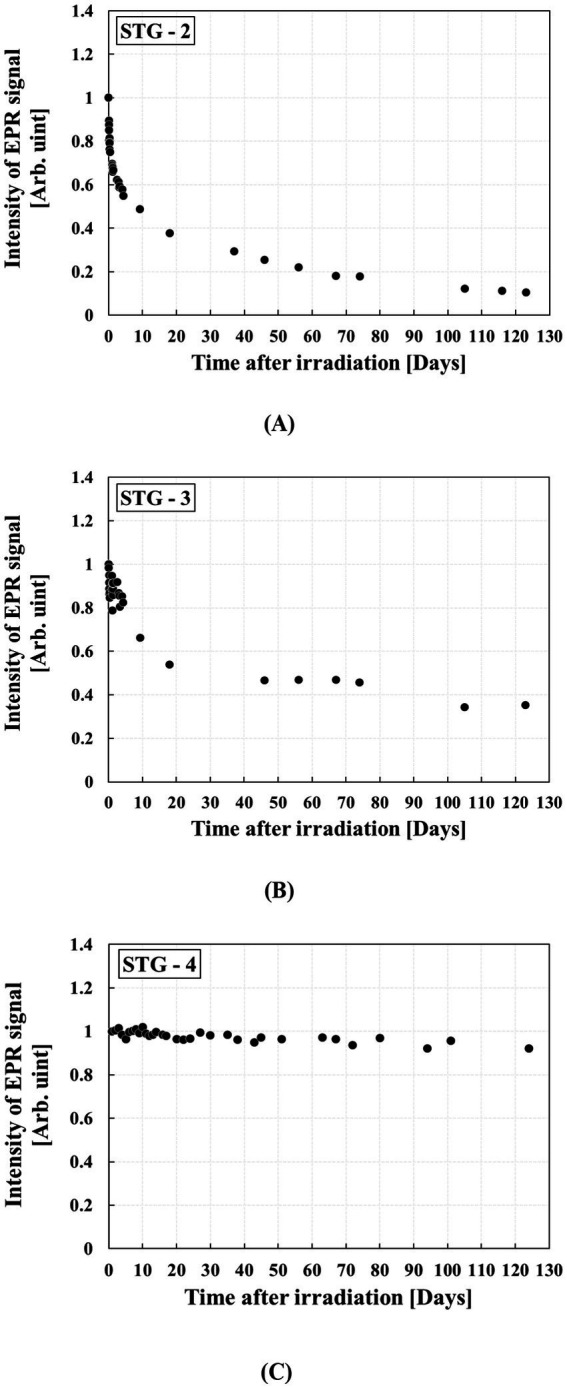
RIS fading in three types of STG over 4 months after irradiation of 20 Gy: **(A)** STG-2, **(B)** STG-3, and **(C)** STG-4.

Figure 5 shows the time stability of RIS for three types of STG. The results indicated distinct differences in RIS time stability among the samples. The RIS intensity decreased by 89.47% in STG-2, 64.71% in STG-3, and 8.48% in STG-4 relative to their initial EPR intensities. While the time stability of RIS for each STG allows for time-dependent dose assessment, STG-4 is a promising candidate for retrospective dosimetry, particularly when the exact time of radiation exposure is unknown.

### 3.3 Dose response

**Figure 6:**
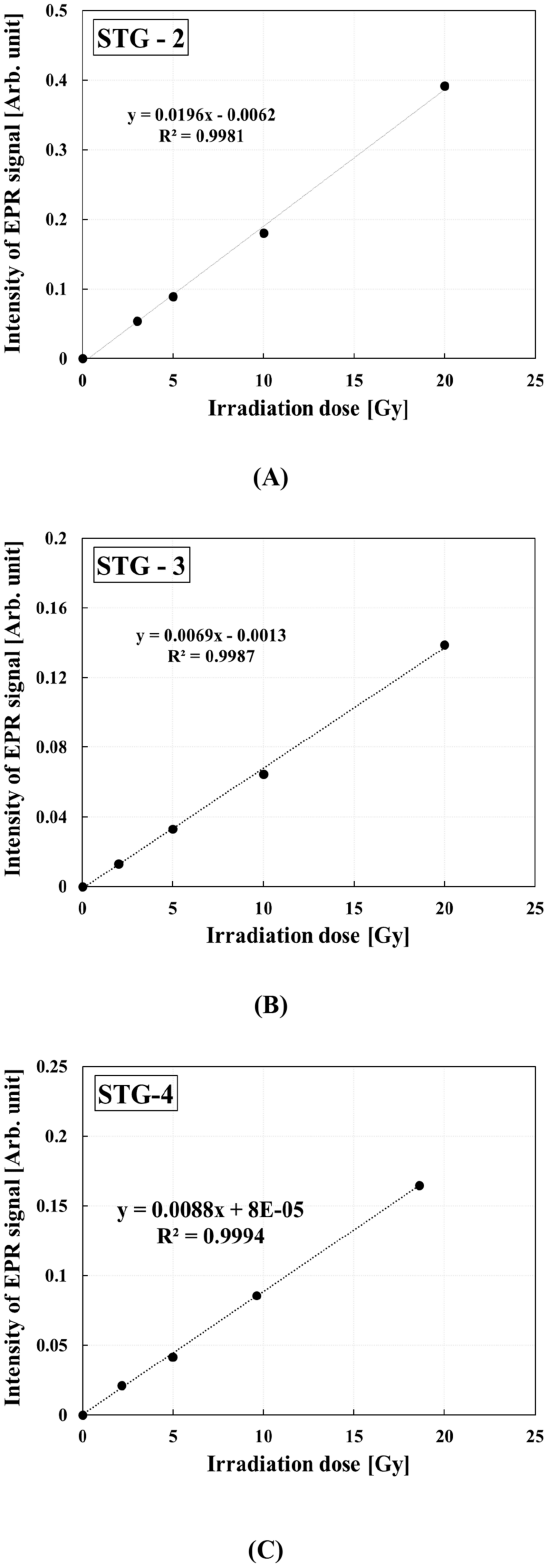
Dose–response curve of STG samples from the three smartwatch models: **(A)** STG-2, **(B)** STG-3, and **(C)** STG-4.

To evaluate the absorbed doses using STGs, analysis of the linear correlation between dose response and radiation dose is required. The dose–response curves of the different STGs are shown in Figure 6. STG-2 showed a clearly distinguishable RIS from the BKS; however, owing to its low RIS stability, its long-term applicability is limited. STG-3 demonstrated minimal differences between RIS and BKS in the EPR spectrum, whereas STG-4 exhibited a distinct linear correlation between irradiation dose and RIS intensity.

### 3.4 Light (UV) effect on RIS

**Figure 7:**
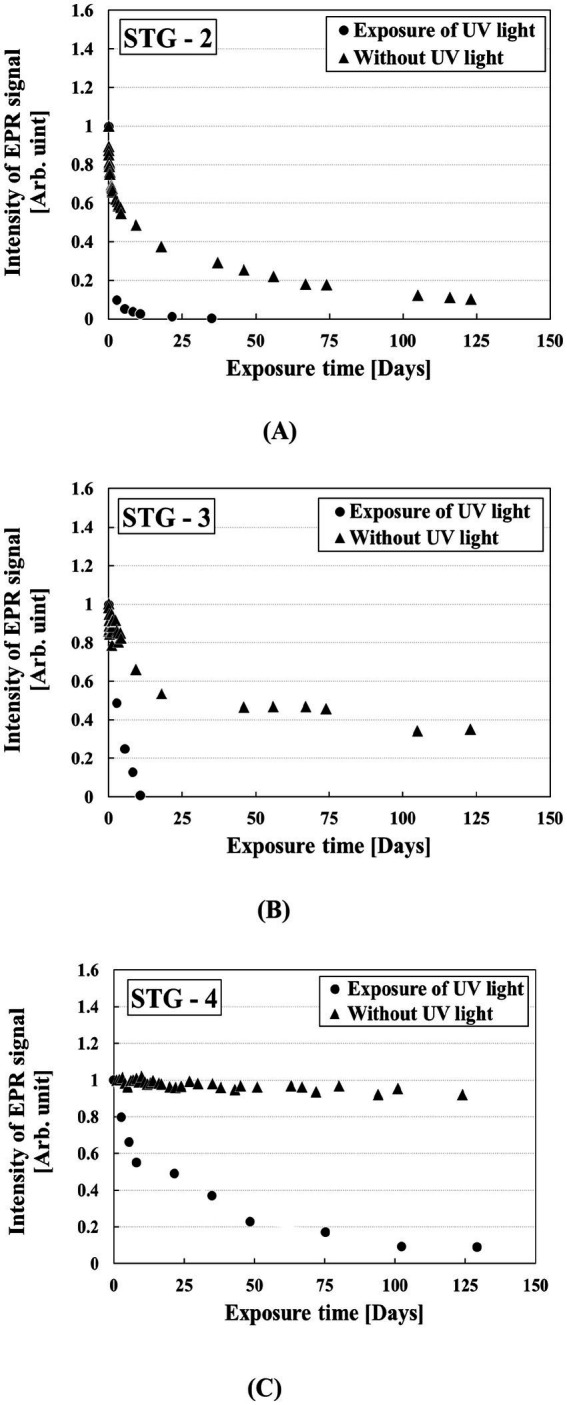
Exposure to UV light and without UV light according to STG samples: **(A)** STG-2, **(B)** STG-3, and **(C)** STG-4.

STG samples were exposed to UV light to evaluate the variation of RIS. Figure 7 shows the RIS variation under UV light and non-UV light exposure. The EPR intensity of RIS under UV light decreased gradually over 35 d for STG-2, 10 d for STG-3, and 100 d for STG-4. The RIS in STG-4 decreased by 87.29% and was evaluated as a residual RIS that was not eliminated by annealing or UV exposure. UV light induced a rapid decrease in RIS compared with the result of time stability of RIS. The attenuation of RIS induced by UV light could lead to underestimation in EPR dosimetry; therefore, the absorbed dose should be calibrated using the RIS fading curve corresponding to UV light exposure.

### 3.5 Thermal effect on RIS

**Figure 8:**
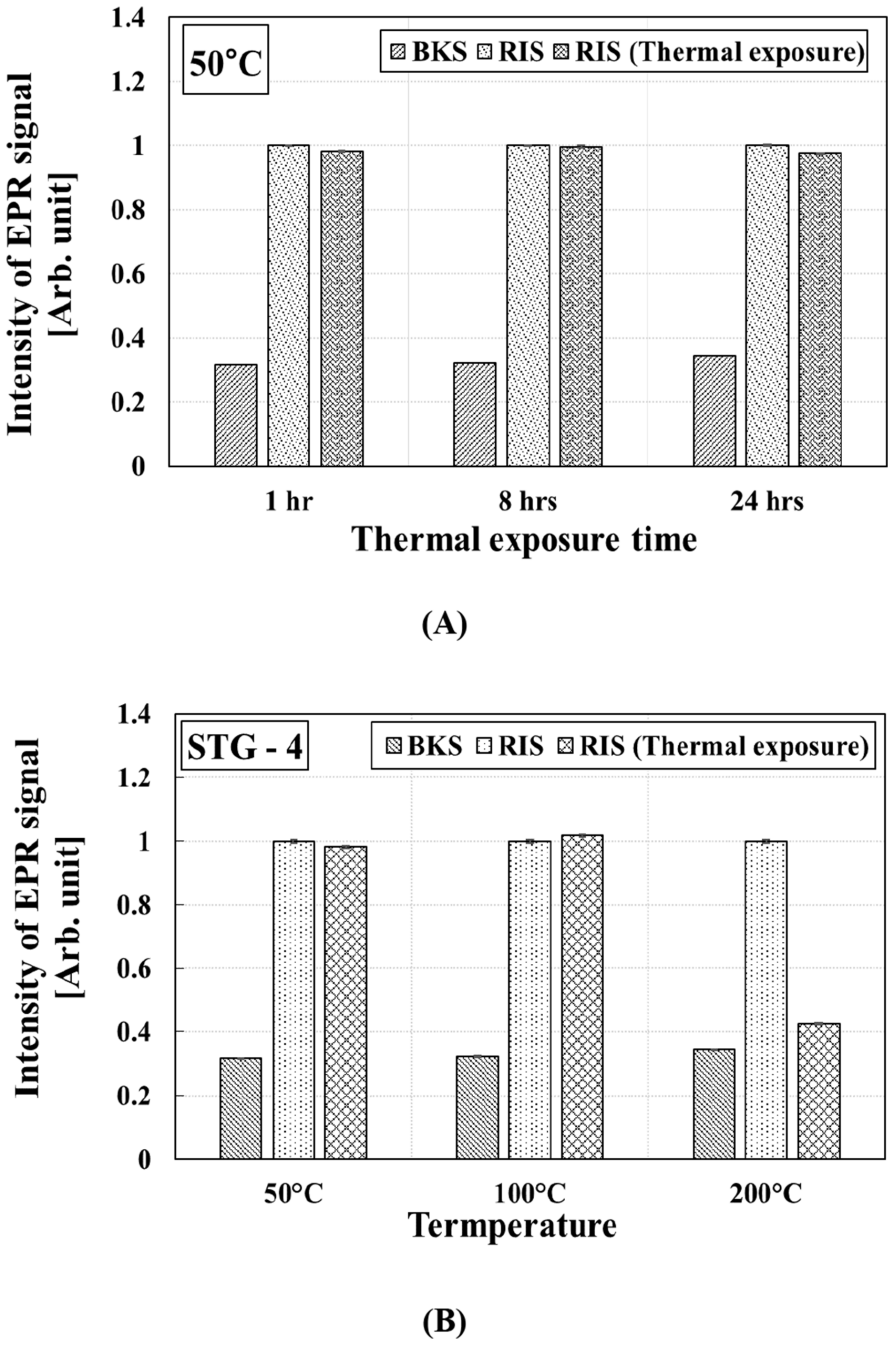
Variation in RIS intensity of STG from STG-4 exposed according to thermal exposure time and temperatures: **(A)** 50 °C for 1, 8, and 24 h and **(B)** 50 °C, 100 °C, and 200 °C for 1 h.

STG-1, STG-2, and STG-3 were excluded from the thermal effect evaluation because no RIS was observed, or the RIS decreased rapidly. STG-4 was used to assess the RIS stability in STGs under thermal exposure. Figure 8 shows the RIS variation with respect to exposure time and temperatures.

As shown in Figure 8A, the RIS variation at 50 °C decreased by 1.77, 0.36, and 2.54% after thermal exposure for 1, 8, and 24 h, respectively. Although RIS showed slight variation at 50 °C, the statistical variation was not significant. The intrinsic temperature of STG-4 during both operation and charging remained below 30 °C. For STG-4, the RIS variation at 50 °C, 100 °C, and 200 °C was −2.59, 2.64%, and −87.74%, respectively. RIS exhibited low variation below 100 °C, whereas a rapid reduction was observed at 200 °C. The thermal effect on RIS in glass is important for BKS estimation in retrospective dosimetry (29).

Additionally, to evaluate the BKS estimation using the thermal effect on RIS, the STG-4 samples were annealed at 200 °C. The signal intensity of STG-4 irradiated with 20 Gy, measured after annealing at 200 °C was approximately 42.73% higher than the initial BKS. The excess signal was attributed to the stable residual RIS that persisted even after thermal treatment. Consequently, a method for BKS estimation accounting for the residual RIS was applied to determine the original BKS of STG-4.

### 3.6 Pretreatment effect on RIS

**Figure 9:**
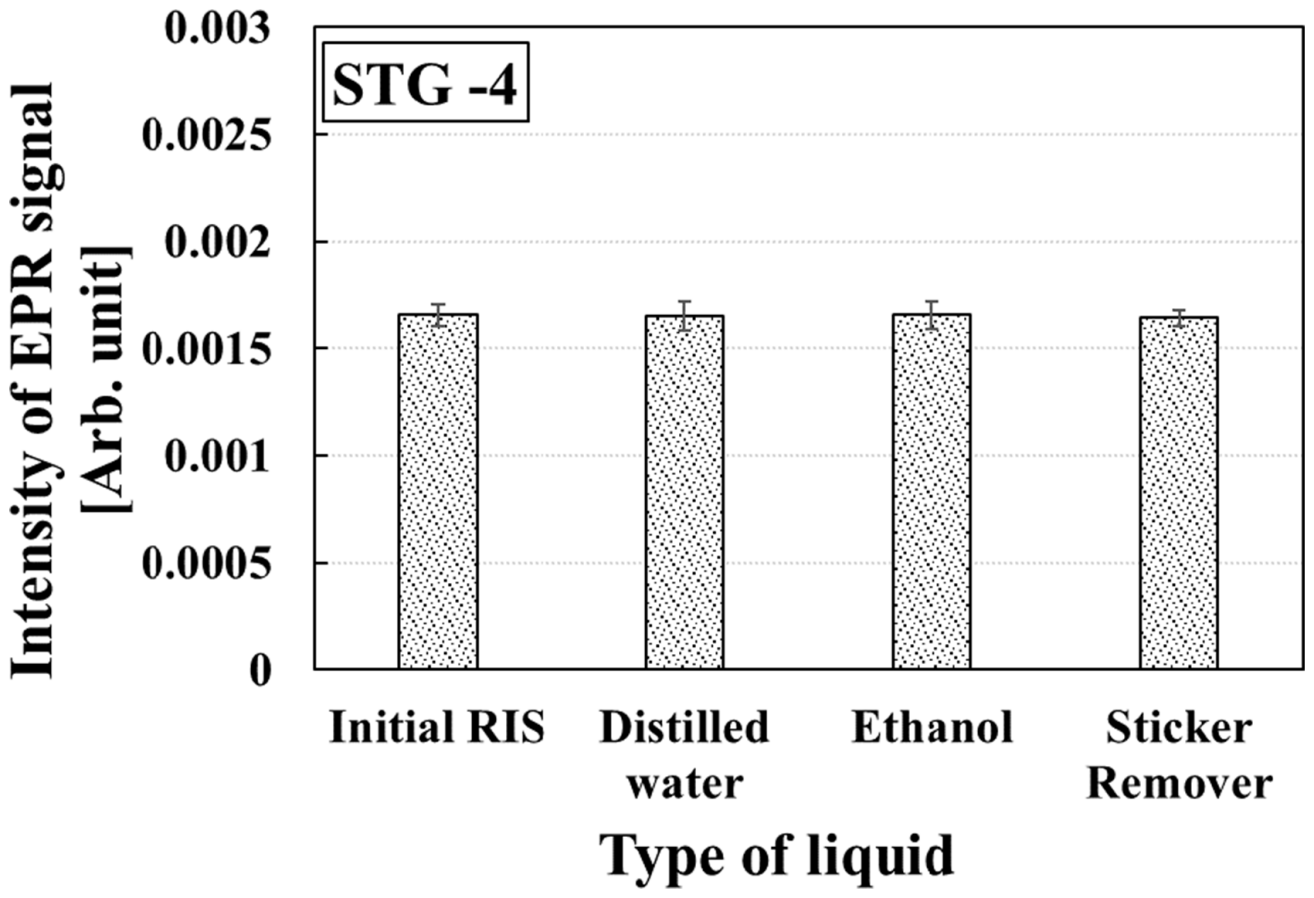
Variation of RIS in STG-4 after pretreatment with distilled water, ethanol, and sticker remover.

For STG-1, STG-2, and STG-3, it was difficult to accurately evaluate the pretreatment effect on RIS due to the absence or fading of RIS in STGs. Therefore, STG-4 was used to evaluate the pretreatment effect on RIS. STG-4 was soaked in distilled water, ethanol, and a commercial sticker remover for 5 min. Figure 9 shows the RIS in STG-4 after 5 min of pretreatment, with changes of −1.23, +2.96%, and −0.82%, respectively.

### 3.7 Minimum detectable dose

STG-4, identified as the most promising STG among the four types, was selected for MDD evaluation. The minimum irradiation dose required to distinguish BKS from RIS in STG-4 was approximately 0.50 Gy. Furthermore, the MDD for STG-4 was determined to be 0.93 Gy. The MDD of STG (EPR) is higher than that of retrospective dosimetry based on tooth (EPR), bone (EPR), resistor (TL), and glass (OSL) (1).

### 3.8 Preliminary STG-EPR dosimetry protocol

The radiological characteristics of the four types of STGs were evaluated for their applicability in assessing doses for individuals exposed during a radiological accident. Among the STGs, STG-4 was selected for the preliminary STG-EPR dosimetry protocol. The distinction between BKS and RIS was clear, and no MIS occurred. The influence of UV light on RIS can be estimated and calibrated, while the reduction of RIS at daily operating temperatures was minimal. In addition, STG-4 exhibited excellent dose response and linearity.

**Figure 10:**
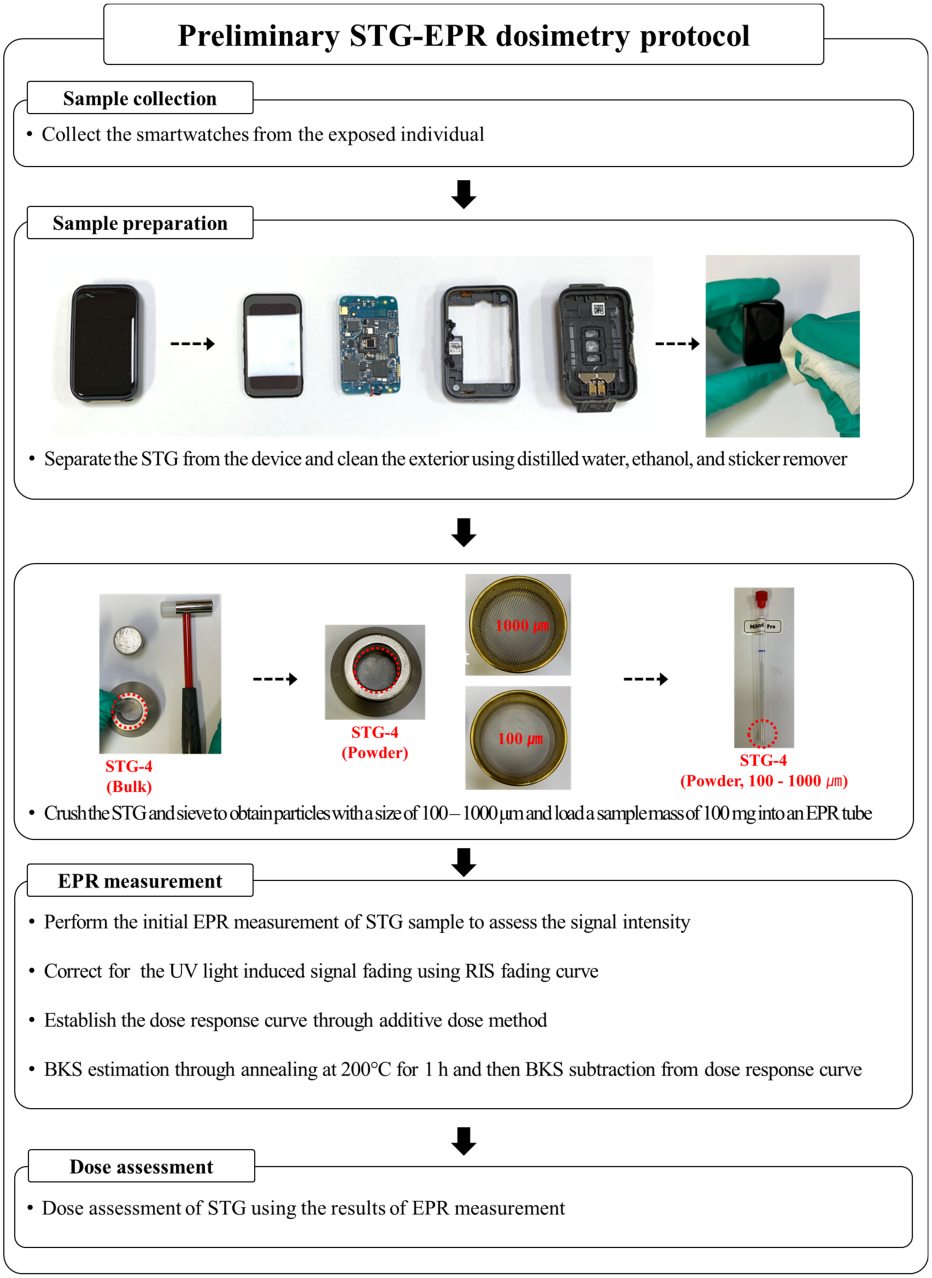
Schematic of preliminary STG-EPR dosimetry protocol.

A schematic overview of the preliminary protocol for STG-EPR dosimetry is shown in Figure 10. The procedure consists of four main phases: sample collection, sample preparation, EPR measurement, and dose assessment. First, in the sample collection phase, the smartwatch is obtained from the exposed individual. During sample preparation, the STG is separated from the smartwatch, and the exterior is cleaned using distilled water, ethanol, and a sticker remover to remove adhesives and surface impurities. The cleaned STG is then crushed and sieved to obtain a uniform particle size of 100–1,000 μm. A sample mass of 100 mg is loaded into an EPR tube for analysis. The EPR measurement phase involves several critical steps. An initial measurement is performed to assess the signal intensity. To ensure accuracy, the protocol applies a correction for signal fading caused by UV light using the established RIS fading curve. A dose–response curve is then constructed using the additive dose method. Additionally, BKS estimation is conducted by annealing the sample at 200 °C for 1 h to measure the residual signal. Finally, in the dose assessment phase, the estimated BKS is subtracted from the total signal to isolate the net radiation-induced signal, and the absorbed dose of STG-4 is determined based on the calibration curve.

To ensure the reliability of the preliminary STG-EPR dosimetry protocol, measurement uncertainty was evaluated in accordance with the Guide to the Expression of Uncertainty in Measurement (GUM) (33).

The uncertainties for pretreatment, time stability of RIS, reproducibility, thermal stability of RIS, BKS estimation, and light (UV) effect were evaluated as Type A uncertainties, derived from the standard deviation of experimental measurements. Specifically, the pretreatment uncertainty was evaluated based on the RIS variation occurring during chemical cleaning and sample crushing. The time stability of RIS was assessed using the variation of RIS measured over 4 months following 20 Gy irradiation. Reproducibility was determined by evaluating the stability of the EPR spectrometer using STG-4 powdered samples. The uncertainty due to thermal stability of RIS was evaluated based on the RIS variation at 50 °C for 24 h. The BKS estimation uncertainty was derived from the variation in radiation dose calculated using the BKS estimation method. The light (UV) effect on RIS was determined from the repeated measurement results of RIS fading after UV exposure equivalent to 0.98 days.

The calibration curve uncertainty was evaluated as a combined uncertainty incorporating the reference irradiation (Type B), mass of the sample (Type A), and regression curve (Type A). The uncertainty for the mass of the sample was derived from mass variations during repeated measurements, while the regression curve uncertainty was evaluated based on the residual standard deviation of the regression analysis (34). The reference irradiation uncertainty was obtained from the calibration of the irradiation system.

The considered contributing factors included pretreatment of the sample, time stability of RIS, reproducibility, thermal stability of RIS, BKS estimation, the light (UV) effect on RIS, reference irradiation, mass of sample, and regression curve. The measurement uncertainty components and their contributions to the STG-EPR dosimetry protocol are summarized in Table 2.

**Table 2:** Measurement uncertainty components and contributions in the preliminary STG-EPR dosimetry protocol.

Contributing factor of uncertainty		Type	Probability distribution	Uncertainty [%]	Contribution [%]
Pretreatment of the sample		A	Normal	1.95	2.74
Time stability of RIS		A	Normal	0.34	0.08
Reproducibility		A	Normal	1.76	2.23
Thermal stability of RIS		A	Normal	0.37	0.10
BKS estimation		A	Normal	4.30	13.31
Light (UV) effect on RIS		A	Normal	8.97	57.90
Calibration curve	Reference irradiation	B	Rectangular	23.30	Rectangular
	Mass of sample	A	Normal	0.20	Normal
	Regression curve	A	Normal	0.15	Normal
Combined uncertainty and contribution				11.79	100
Expanded uncertainty at confidence level of approximately 95%, *k* = 2				23.58	

As shown in Table 2, the most significant contribution to the overall uncertainty was from the light (UV) effect on RIS, which was evaluated as 8.97% and accounted for 57.90% of the combined uncertainty. The uncertainties of the calibration curve and BKS estimation were 5.73 and 4.30%, accounting for 23.65 and 13.31% of the combined uncertainty, respectively.

### 3.9 Blind test of the preliminary STG-EPR dosimetry protocol

**Figure 11:**
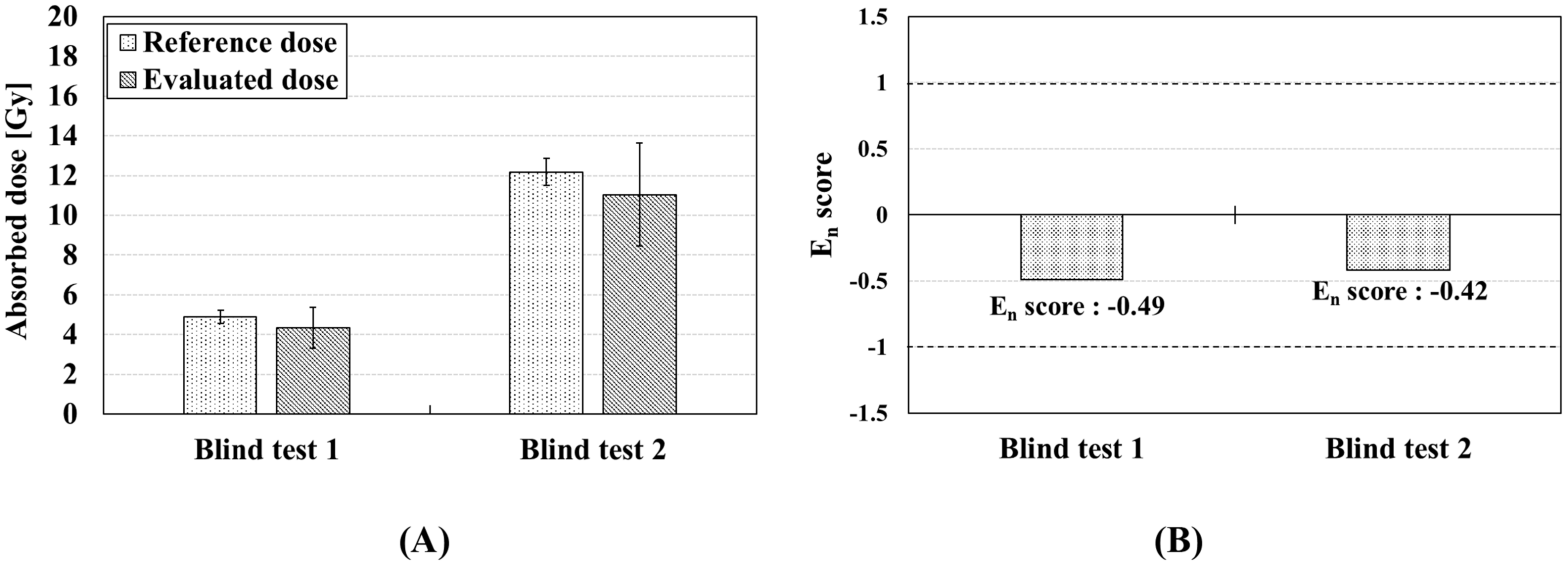
Results of blind test using preliminary STG-EPR dosimetry protocol: **(A)** reference and evaluated doses of STG samples from STG-4 and **(B)** E_n_ scores for the blind tests.

To assess the preliminary STG-EPR dosimetry protocol, a blind test was conducted using two smartwatches containing STG-4. As shown in Figure 10, the absorbed doses of the two samples were determined to be 4.35 ± 1.02 Gy and 11.04 ± 2.60 Gy, respectively. The corresponding reference doses were 4.88 ± 0.32 Gy and 12.18 ± 0.69 Gy. The absorbed doses of STG-4 were underestimated compared with the reference doses. The difference between reference and evaluated doses is considered to be predominantly attributable to the UV light effect on RIS and BKS estimation. Overall, the E_n_ scores for blind tests 1 and 2 were −0.49 and −0.42, respectively, which are within the acceptance criterion of ± 1 (Figure 11).

## 4 Conclusion

Several components of smartwatches have been evaluated as potential materials for retrospective dosimetry in radiological accidents, as smartwatches are consistently worn on the body and are widely available. To develop STG-based retrospective dosimetry, the EPR technique was used to evaluate radiological characteristics such as BKS, RIS, MIS, LIS, dose–response, light (UV) effect on RIS, thermal and pretreatment effects on RIS, and MDD. Among the evaluated STGs, STG-4 exhibited a clear RIS and demonstrated promising radiological characteristics in terms of dose–response, dose linearity, time stability of RIS, and minimal RIS variation under thermal and pretreatment conditions. A blind test was conducted using the preliminary protocol of STG-EPR dosimetry based on the radiological characteristics of STG-4. The E_n_ score was calculated using the evaluated absorbed dose, reference dose, and their associated measurement uncertainties. Under laboratory conditions, the blind test results demonstrated the potential of STG-EPR dosimetry for radiological incident scenarios. Furthermore, STG-EPR dosimetry can be useful not only in general radiological incidents but also in localized hand exposure scenarios involving relatively high-dose exposure incidents. Differences between laboratory and real-world scenarios are mainly due to the difficulty in accurately determining the UV light exposure. The preliminary STG-EPR dosimetry protocol is considered applicable when the smartwatch has not been exposed to UV light or when the precise UV light fluence is known. However, in the absence of accurate information on UV light exposure, the applicability of the protocol is limited. Since UV light exposure time has a significant impact on the accuracy and uncertainty of dose assessment, further studies are required to establish the correlation between UV light and RIS (20). Moreover, the dosimetric characteristics of STG are dependent on their material composition. The results of dosimetric characteristics cannot be generalized to all STGs. Additionally, due to variability in STG materials and the lack of comprehensive radiological characterization for all smartwatch models, the applicability of the developed protocol may be limited to STGs with evaluated radiological characteristics. Therefore, further research is needed to evaluate the radiological characteristics of various smartwatch models and to accommodate the continued release of new devices.

## Data Availability

The original contributions presented in the study are included in the article/supplementary material, further inquiries can be directed to the corresponding author.
